# Molecular Detection of the -308 G/A Polymorphism in the *Tumor Necrosis Factor Alpha* (*TNFα*) Gene Promoter and Its Relationship with the Development of Severe Dengue in Patients from Urabá Subregion, Antioquia-Colombia

**DOI:** 10.3390/microorganisms14102354

**Published:** 2026-10-14

**Authors:** Jorge Emilio Salazar Flórez, Ronald Guillermo Peláez Sánchez, Luz Stella Giraldo Cardona, Leci Camila Ariza Salas, Juliana María Martínez Garro, Marco Torres-Castro, Fernando P. Monroy, Luis Ernesto López-Rojas, Katerine Marín Velasquez, Berta Nelly Restrepo Jaramillo, Margarita Arboleda Naranjo

**Affiliations:** 1 GEINCRO Research Group, School of Health Sciences, San Martin University, Sabaneta 055457, Colombia; ronald.pelaez@sanmartin.edu.co (R.G.P.S.); luz.giraldo@sanmartin.edu.co (L.S.G.C.); 2 CES Biology Research Group, Faculty of Agricultural and Natural Sciences, CES University, Medellín 050021, Colombia; ariza.leci@uces.edu.co (L.C.A.S.); jmartinezg@ces.edu.co (J.M.M.G.); 3 Dr. Hideyo Noguchi Regional Research Center, Laboratory of Zoonoses and Other Vector-Borne Diseases, Autonomous University of Yucatan, Mérida 97000, Mexico; antonio.torres@correo.uady.mx; 4 Department of Biological Sciences, Northern Arizona University, Flagstaff, AZ 86011, USA; fernando.monroy@nau.edu; 5 Colombian Institute of Tropical Medicine, CES University, Medellín 050021, Colombia; lelopez@ces.edu.co (L.E.L.-R.); kmarin@ces.edu.co (K.M.V.); nellyrpo@hotmail.com (B.N.R.J.); marboleda@ces.edu.co (M.A.N.)

**Keywords:** dengue virus, severe dengue, TNFα, genetic polymorphism, Urabá subregion, Antioquia, Colombia

## Abstract

Dengue fever is a viral infection caused by the dengue virus (DENV), a positive sense RNA virus belonging to *Flaviviridae* family. The virus is transmitted to humans through the bite of the *Aedes aegypti* mosquito. Worldwide, between 100 and 400 million cases are reported annually. The infection in humans can cause three clinical forms, which are related to severity: dengue without warnings signs, dengue with warnings signs, and severe dengue. Genetic variants in genes encoding immune system proteins have been associated with severe clinical forms of dengue, including variants in *TNF-α*. *TNF-α* plays a key role in dengue immunopathogenesis, and promoter polymorphisms may influence *TNF-α* expression and inflammatory responses associated with severe disease. Therefore, understanding host genetic variation may contribute to elucidating mechanisms involved in progression to severe clinical forms. This study explored the association between the *TNF-α* -308 G>A promoter polymorphism and dengue severity. We included 92 laboratory-confirmed dengue cases: 30 with dengue without warning signs, 43 with dengue with warning signs, and 19 with severe dengue. Genotyping was performed using PCR amplification, Sanger sequencing, and bioinformatic analysis of the *TNF-α* promoter region. Because of the low frequency of A allele carriers and sparse data, pairwise Firth penalized logistic regression models adjusted for age and primary/secondary infection status were used to evaluate the association between variant carrier status and dengue severity. The *TNF-α* -308 A allele was detected in a small proportion of participants, with variant carriers accounting for 10.5% of severe dengue cases. Adjusted analyses showed higher point estimates for severe dengue compared with dengue without warning signs and dengue with warning signs; however, the associations were not statistically significant, and confidence intervals were wide. Genotype frequencies showed no statistically significant deviation from Hardy–Weinberg equilibrium (*p* = 0.112), and the estimated post hoc statistical power was 11.3%. The *TNF-α* -308 G>A polymorphism was not significantly associated with dengue severity in this sample. The observed estimates were imprecise and should be considered exploratory, warranting confirmation in larger and more diverse populations.

## 1. Introduction

Dengue fever is a viral infection caused by the dengue virus (DENV), a positive sense RNA virus belonging to *Flaviviridae* family. DENV has four distinct serotypes (DENV-1 to DENV-4). The virus is transmitted to humans through the bite of the *Aedes aegypti* mosquito [1]. Worldwide, dengue virus infection has been described in more than 100 countries; between 100 and 400 million cases are reported annually, and approximately 5.6 billion people are at risk of infection (around half of the world’s population) [2]. From 2014 to 2022, Brazil and Colombia accounted for 84% of South American cases; in 2022, Colombia reported 69,497 cases, including 1371 severe instances and 48 deaths, with Antioquia department being heavily affected, particularly in the Chigorodó (14.6% cases), and Turbo (12.5% cases) municipalities [3,4], highlighting the endemic behavior of dengue in the Urabá subregion. When the infection occurs in humans, the disease can present in three clinical forms related to severity: dengue without warnings signs (DWOS), dengue with warnings signs (DWWS), and severe dengue (SD), the most critical clinical form characterized by plasma leakage, shock, and organ failure [5]. Prevention and control strategies are based on chemical and biological control of the vector, supportive clinical treatment of the patients, and community education to prevent the vector’s multiplication cycle [6]. Additionally, there is no specific treatment for the infection, and the vaccines developed are not widely used (Dengvaxia^®^ CYD-TDV, and Qdenga^®^ TAK-003) [7]. There are several gaps in knowledge about dengue infection that limit the development of more effective treatments [8], universal vaccinations [9], and control strategies [9], including: incomplete immunopathogenesis [10], host genetic variability [11], molecular mechanisms of the virus to cause infection [12], specific antiviral treatments [7], insecticide resistance in the mosquito vector [13], and the impact of climate change on the epidemiological patterns of disease transmission [14]. Therefore, the present research focuses on the study of the immunopathogenesis of dengue virus infection. The immunopathogenesis of the dengue virus refers to the mechanisms by which the host’s immune response, instead of merely controlling the infection, contributes to tissue damage and progression to severe forms such as dengue hemorrhagic fever and dengue shock syndrome [15,16]. Some key aspects for studying immunopathogenesis are: antibody dependent enhancement (ADE) [12], cytokine storm [17], immune evasion [10], and host genetic factors [11,18]. In this study we will focus on host genetic factors that may contribute to differences in clinical severity among individuals with dengue infection. Among these factors, tumor necrosis factor alpha (*TNF-α*) plays a diverse role in cellular differentiation, regulation, proliferation, inflammation, and the modulation of both innate and adaptive immune responses and has been implicated in various infectious and autoimmune diseases [19,20,21]. *TNF-α* has been associated with severe dengue through mechanisms involving endothelial cell activation, induction of inflammatory mediators, survival of inflammatory cells, production of tissue destructive enzymes, and apoptosis [19,20,21]. Genetic variants in the *TNF-α* gene, particularly promoter polymorphisms, have been associated with altered *TNF-α* expression and inflammatory responses potentially involved in severe forms of dengue [22,23,24,25,26,27,28]. Therefore, the objective of the present study was to characterize the *TNF-α* -308 G>A promoter polymorphism and evaluate its association with dengue severity among laboratory-confirmed dengue patients from the Urabá subregion (Antioquia, Colombia).

## 2. Materials and Methods

### 2.1. Study Sites and Samples

A retrospective observational genetic association study was conducted among patients with laboratory-confirmed dengue from the Urabá subregion (Antioquia, Colombia) between January 2018 and December 2022. In this study, we focused on host genetic factors that may contribute to differences in clinical severity among individuals with dengue infection. The patients selected for the study met the following inclusion criteria: clinical symptoms and a positive diagnosis for dengue, confirmed through one of the following methods: (1) positive DENV specific real-time reverse transcription polymerase chain reaction (RT-PCR); (2) confirmation of the dengue virus NS1 antigen using capture ELISA on acute phase serum samples; and (3) detection of dengue virus specific IgM or IgG antibodies via ELISA. Cases were classified in accordance with the World Health Organization’s (WHO) 2009 guidelines for dengue: dengue with warnings signs, dengue without warnings signs, and severe dengue [5]. DWOS was used as the reference clinical group for pairwise comparisons and was not considered an uninfected control group. The patients who agreed to participate in the study signed the informed consent, and the blood samples for DNA extraction were collected in tubes with EDTA as an anticoagulant.

### 2.2. Sociodemographic and Clinical Data Collection

A clinical data collection form was developed to categorize laboratory-confirmed dengue cases. Data were compiled from primary sources through interviews with each patient and secondary source via medical record reviews. Collected sociodemographic data included age, gender, ethnicity, education level, occupation, socioeconomic status, insurance type, and place of origin. Clinical data encompassed disease type, comorbidities, medical history, symptoms, and laboratory results (specifically blood counts), and liver and renal function tests alongside a documentation of the symptom onset and progression of disease severity within the first 5 to 7 days of disease manifestation. Potential coinfections with other pathogens such as *Leptospira*, *Zika*, and *Chikungunya* were also investigated. The clinical data were extracted within the first five days of the disease’s progression.

### 2.3. DNA Extraction and Quantification

Genomic DNA was extracted from 200 µL of blood collected from dengue patients using the QIAamp DNA Mini Kit (QIAGEN^®^, Hilden, Germany), which utilizes silica columns for DNA adsorption. The extraction followed the manufacturer’s protocol for peripheral blood samples. Initially, blood cell lysis was achieved by mixing 20 µL of QIAGEN protease (QP), 200 µL of the blood sample, and 200 µL of lysis buffer (AL), followed by the addition of 200 µL ethanol to the mixture. The lysate was then applied to a silica column and centrifuged to bind the genomic DNA. This was followed by two washing and purification steps, the first using 500 µL of Wash Buffer 1 (AW1) and centrifugation, and the second with 500 µL of Wash Buffer 2 (AW2). The genomic DNA was finally eluted in 50 µL of Elution Buffer (AE). The concentration of DNA extracts from the patients was determined using a NanoDrop 1000 spectrophotometer (Thermo Scientific, Waltham, MA, USA), with the elution buffer from the QIAamp DNA Mini Kit serving as a blank. For each measurement, 2 µL of the DNA sample was used. DNA integrity was further assessed by electrophoresis on 2% agarose gels.

### 2.4. TNF-α Gene Promoter Amplification and Visualization

The PCR protocol was optimized to identify conditions that would yield the best amplification of a 107 base pair fragment of the *TNF-α* gene promoter, using primers Forward-proTNF (AGGCAATAGGTTTTGAGGGCCAT) and Reverse-proTNF (TCCTCCCTGCTCCGATTCCG) described by Wilson et al. [29]. The optimized PCR reaction mix included 0.4 µM of each oligonucleotide, 0.2 mM dNTPs, 1× PCR buffer, 1.5 mM MgCl_2_, 1 unit of Taq polymerase, and 20 ng/µL of DNA, with a final reaction volume of 25 µL. Amplification was carried out in a C1000 Touch thermocycler (BioRad, Hercules, CA, USA) with the following thermal conditions: an initial denaturation at 94 °C for 5 min; 35 cycles of 94 °C for 45 s, 63 °C for 1 min, and 72 °C for 1 min; followed by a final extension at 72 °C for 5 min. Electrophoresis was conducted immediately after PCR amplification to visualize the products. A mixture of 5 μL of the PCR product and 5 μL of running buffer was loaded onto a 2% agarose gel, prepared with Condalab agarose E. Alongside, 10 μL of a 50 bp DNA ladder (50–1000 ExcelBand™ 50 bp DNA Ladder, SMOBIO Technology, Inc, Hsinchu City, Taiwan) was used as a molecular weight marker. The gel was run in 500 mL of 1× TBE buffer (containing 40 mM Tris-Borate and 1 mM EDTA) at a constant 90 volts for 45 min. Post electrophoresis, the gel was stained with 5 μL of EZBlue™ Gel Staining Reagent for visualization. The results were observed under a GelDoc ultraviolet light transilluminator (BioRad, Hercules, CA, USA) and captured using Quantity One version 4.6 software.

### 2.5. Sequencing, Variant Characterization, and Evolutionary Analysis of the TNF-α Gene Promoter

The 107 base pair fragments of the *TNF-α* gene promoter, amplified from the samples, were sent to Macrogen^®^ Korea for sequencing. Utilizing the Sanger sequencing method paired with capillary electrophoresis (CES) on an ABI 3730xl System (Thermo Scientific, Waltham, MA, USA) and managed via LIMS software version 21.3, sequencing was performed. Each sample was sequenced in both forward and reverse directions to ensure accuracy, with a consensus sequence generated using Mega software version XII. The reference sequence for the *TNF-α* gene promoter located in the 5’UTR was downloaded from the NCBI database (accession number NCBI: LC775561.1). Employing the MEGA XI bioinformatics suite, the promoter sequence was analyzed to determine the primer amplification region for both forward and reverse primers. The nucleotide position -308 within the reference sequence was identified for analysis of the *TNF-α* -308 G>A polymorphism. Consensus sequences from all 92 patients were aligned against the reference sequence, and the nucleotide present at position -308 was used to classify participants into three genotypes: GG homozygous, GA heterozygous, and AA homozygous. The distributions of genotype frequencies in the patient groups (SD: severe dengue, DWWS: dengue with warnings signs, and DWOS: dengue without warnings signs) were calculated. Finally, Allele frequencies of the *TNF-α* -308 G/A polymorphism were calculated by direct allele counting from the observed genotypes (GG, GA, and AA) in 92 dengue patients. Hardy–Weinberg equilibrium (HWE) was assessed using the exact Hardy–Weinberg test, which is recommended for variants with low minor allele frequencies and small expected genotype counts. A *p*-value < 0.05 was considered statistically significant. To evaluate the evolutionary conservation of the *TNF-α* gene promoter, the following nucleotide sequences were downloaded from the NCBI database using these accession numbers: *Homo sapiens* (L11698.1), *Pan troglodytes* (AB210166.1), *Gorilla Gorilla* (CU104666.2), *Pan paniscus* (DQ482190.1), *Pongo abelii* (AC206710.3), *Hylobates lar* (AF195666.1), and *Macaca mulatta* (FJ215749.1). Sequence alignment and phylogenetic tree reconstruction were performed using the bioinformatics software MEGA version 12, with the following configuration parameters: The evolutionary history was inferred using the Neighbor-Joining method. The optimal tree with the sum of branch length = 0.096 is shown. The percentage of replicate trees in which the associated taxa clustered together in the bootstrap test (1000 replicates) is shown next to the branches. The tree is drawn to scale, with branch lengths in the same units as those of the evolutionary distances used to infer the phylogenetic tree. The evolutionary distances were computed using the Kimura-2 parameter method and are in the units of the number of base substitutions per site. The rate variation among sites was modeled with a gamma distribution (shape parameter = 1.00). The analytical procedure encompassed 7 nucleotide sequences. The pairwise deletion option was applied to all ambiguous positions for each sequence pair, resulting in a final data set comprising 1.189 positions. Evolutionary analyses were conducted in MEGA12 utilizing up to 8 parallel computing threads. To identify transcription factor recognition sites, the JASPAR web server (https://jaspar.elixir.no/) was used, directing the search to Vertebrata (*Homo sapiens*) database and a relative profile score threshold of (85%). The impact of the rs1800629 polymorphism on the TFAP2A transcription factor recognition site was plotted using Mega Version 12.1^®^ (https://www.megasoftware.net, accessed on 14 September 2026) and Microsoft Office—PowerPoint^®^ (https://www.microsoft.com/es-co/microsoft-365/microsoft-office, accessed on 14 September 2026).

### 2.6. Statistical Analysis

Statistical analyses were conducted using the R software package, version 4.3.2, available at https://cran.r-project.org/. To assess the differences in frequencies of demographic, clinical, laboratory, and genotype variables among dengue patient groups (SD, DWWS, and DWOS), the chi-square test (χ^2^) or Fisher’s exact test was employed. Continuous variables were assessed for normality within each clinical group using the Shapiro–Wilk test. For consistency of presentation, continuous laboratory variables were summarized using the median and interquartile range (IQR). Between group comparisons were performed using one-way ANOVA for variables meeting the normality assumption and the Kruskal–Wallis test otherwise. All statistical tests were two sided, and *p*-values < 0.05 were considered statistically significant. Given the low frequency of the *TNF-α* -308 mutant genotype and the presence of sparse cells, pairwise penalized logistic regression models using Firth’s correction were fitted to reduce small sample bias. Mutant carrier status was defined as the presence of at least one A allele (GA/AA), with the GG genotype as the reference category. Three pairwise comparisons were evaluated: severe dengue (SD) versus dengue without warning signs (DWOS), severe dengue versus dengue with warning signs (DWWS), and DWWS versus DWOS. For each comparison, the first clinical group was coded as the outcome category. Models were adjusted a priori for age and primary/secondary infection status as clinically relevant covariates. Age was modeled per 10-year increase. Adjusted odds ratios (aORs) with 95% confidence intervals (CIs) were reported. DENV serotype was not included in the adjusted models because this information was unavailable for a substantial proportion of participants. Clinical manifestations and complications related to dengue severity were not included as covariates because they were considered components or consequences of the outcome rather than baseline confounders. As a sensitivity analysis, severe dengue was additionally compared with the combined non-severe group (DWOS and DWWS). To evaluate the ability of the study to detect an association between the *TNF-α* -308 G/A polymorphism and dengue severity, a post hoc statistical power analysis was performed. Power was estimated using a two-sided comparison of proportions with a significance level of 0.05. The analysis was based on the observed frequency of *TNF-α* -308 A allele carriers (GA/AA) among patients with severe dengue and those with non-severe dengue. The R/Quarto code used for the statistical analyses has been deposited in a public GitHub repository to enhance transparency and reproducibility. The repository is available at: https://github.com/lstellagiraldo-dotcom/TNFa-dengue-Uraba (accessed on 26 September 2026).

## 3. Results

### 3.1. Study Sites and Samples

**Figure 1:**
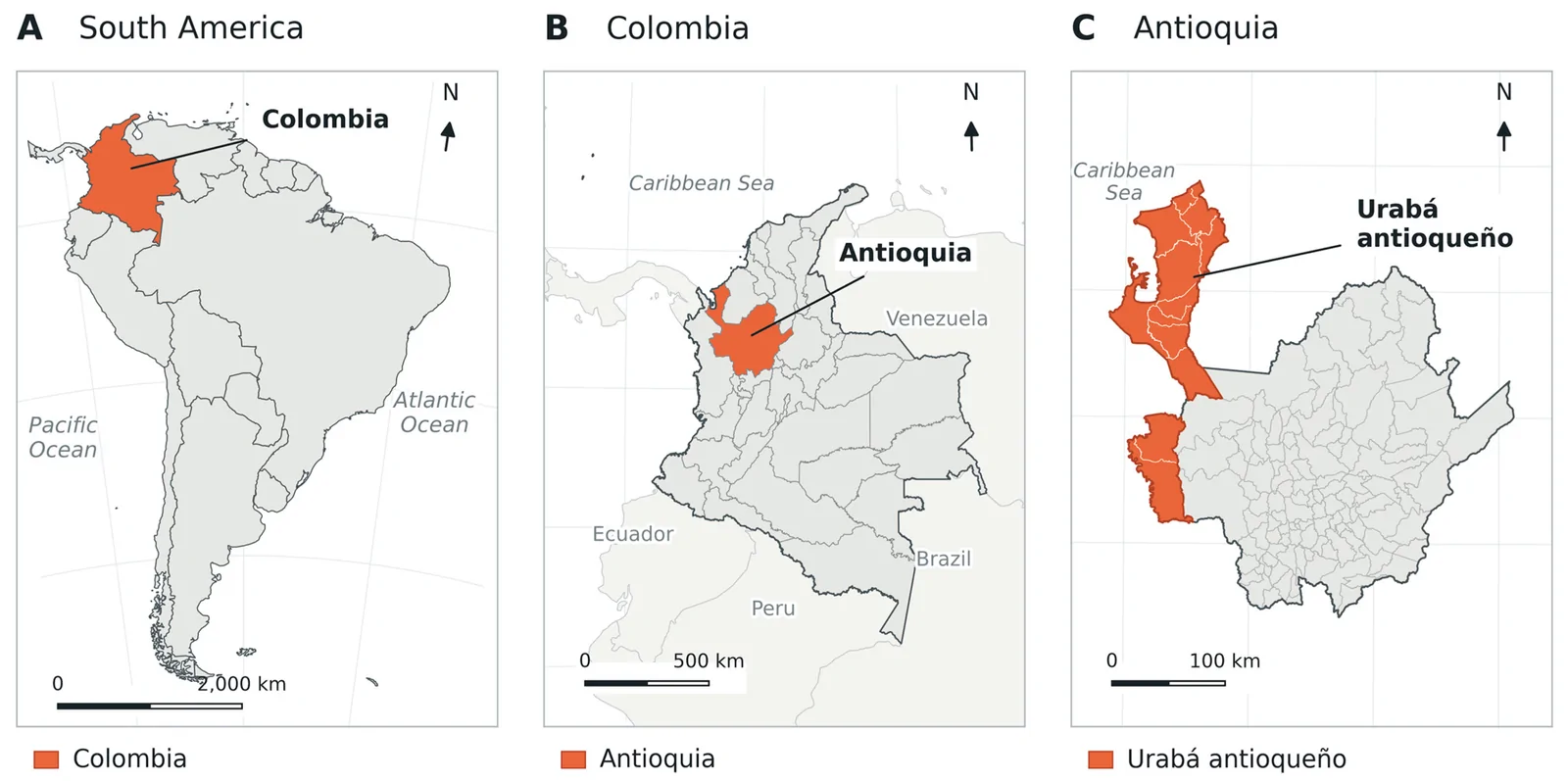
The map shows the geographic location of Colombia, Antioquia department, Urabá subregion, and the municipalities of Apartadó, Arboletes, Carepa, Chigorodó, Murindó, Mutatá, Necoclí, San Juan de Urabá, San Pedro de Urabá, Turbo, and Vigía del Fuerte. The red color indicates the country (Colombia), department (Antioquia), and subregion (Urabá). Maps were generated in Python from Natural Earth 1:50 million data, version 5.1.1 (public domain), and geoBoundaries gbOpen Colombia layers: ADM1, representing 2017 (OpenStreetMap/Wambacher; © OpenStreetMap contributors, ODbL 1.0; https://www.openstreetmap.org/copyright (accessed on 26 September 2026), and ADM2, representing 2020 (DANE; CC BY 4.0, https://creativecommons.org/licenses/by/4.0/ (accessed on 26 September 2026). Boundaries were reprojected for display; Antioquia and Urabá outlines in panel (**C**) were obtained by dissolving municipal polygons. Subregion membership follows the Gobernación de Antioquia.

The Antioquia department is located in northwestern Colombia. Its geographical coordinates extend between 05°26′20″ and 08°52′23″ North latitude, and between 73°53′11″ and 77°07′16″ West longitude of the Greenwich meridian, and it is divided into nine subregions (Urabá, Bajo Cauca, Magdalena Medio, Nordeste, Norte, Occidente, Oriente, Suroeste, and Valle de Aburrá) and 125 municipalities. The Urabá subregion is geographically located at the central coordinates (7°52′59.99″ N, 76°37′59.88″ W), and it is made up of 11 municipalities: Apartadó, Arboletes, Carepa, Chigorodó, Murindó, Mutatá, Necoclí, San Juan de Urabá, San Pedro de Urabá, Turbo, and Vigía del Fuerte. A total of 333 patients with a clinical diagnosis of dengue were identified through active community outreach and research promotion activities conducted through healthcare institutions. However, only 92 patients agreed to participate in the study and signed the informed consent form. The 92 patients come from the following municipalities: Apartadó (60 patients, 65.21%), Turbo (20 patients, 21.73%), Carepa (seven patients, 7.60%), and Chigorodó (five patients, 5.43%) (Figure 1), including patients from 4/11 Urabá subregion municipalities. Among the 92 patients with a positive diagnosis for dengue, 30 patients (32.6%) were classified as dengue without warnings signs, 43 patients (46.7%) as dengue with warnings signs, and 19 patients (20.6%) as severe dengue. All patients agreed to donate a peripheral blood sample (5 mL) for the research (Table 1 and Table S1).

### 3.2. Sociodemographic and Clinical Data Collection

The study analyzed the -308 *TNF-α* gene promoter polymorphism in 92 dengue patients, with an average age of 13.0 years (SD = 12.8). The demographic analysis revealed no significant differences between the groups (*p*-value > 0.05). Most participants were male, residing in urban areas, and affiliated with a contributory healthcare regime. The 80% of patients in both the DWWS and DWOS groups had achieved an elementary or secondary level of education (Table 1). Clinical characteristics varied among dengue patient groups (DWOS, DWWS, and SD), as summarized in (Table 2). There were no significant differences in the presence of comorbidity, infection type, and coinfection among the groups. However, most signs and symptoms showed statistically significant differences between the patient groups, except for extremities edema. The SD group exhibited a higher prevalence of symptoms such as abdominal pain, nausea, fluid accumulation, ascites, irritability, hepatomegaly, splenomegaly, and hematemesis (over 20%) compared to the DWWS and DWOS groups. The DWWS group reported a higher frequency of symptoms, including retro orbital pain, fatigue, dizziness, arthralgia, and pruritus, with a prevalence exceeding 50% compared to the SD and DWOS groups. The DWOS group reported a higher frequency of symptoms, including myalgia and exanthem, with a prevalence exceeding 50% compared to the SD and DWWS groups. Statistically significant differences were observed in laboratory findings across the groups. The median platelet count, percentage of eosinophils, and percentage of basophils were notably lower in patients with SD compared to other groups (*p*-value < 0.05). The median hematocrit, white blood cells, creatinine, blood urea nitrogen (BUN), and activated partial thromboplastin time (aPTT) were notably higher in patients with SD compared to other groups (*p*-value < 0.05). Heart rate and hemoglobin levels also differed significantly among the groups (*p*-value < 0.05), with higher median values observed in the SD group. In contrast, patients in the DWWS group had intermediate values for several laboratory parameters compared to the DWOS and SD groups, suggesting greater hematological and biochemical alterations with increasing disease severity (Table 3 and Table S1).

### 3.3. DNA Extraction and Quantification

DNA was extracted from 92 peripheral blood samples from dengue patients. Sample quality was assessed by electrophoresis on a 1.5% agarose gel, and all 92 samples showed good genetic integrity, with no degradation observed. Protein contamination was tested on all 92 samples using the 260/280 ratio, yielding values between 1.89 and 2.03, highlighting the purity of the DNA. Salt, alcohol, and phenol contamination was also assessed using the 260/230 ratio, with values between 1.9 and 2.08, confirming the purity of the DNA. DNA concentrations ranged from 30.6 to 244.5 ng/µL; these concentrations were sufficient for amplification and sequencing of a 107 base pairs fragment of the *TNF-α* gene promoter.

### 3.4. TNF-α Gene Promoter Amplification and Visualization

The endpoint polymerase chain reaction (PCR) was standardized, successfully amplifying a specific band of 107 base pairs from the DNA samples of the 92 patients analyzed. The negative controls showed no contamination, and the positive controls amplified satisfactorily, demonstrating proper functioning of the PCR (Figure S1).

### 3.5. Sequencing, Variant Characterization, and Evolutionary Analysis of the TNF-α Gene Promoter

**Figure 2:**
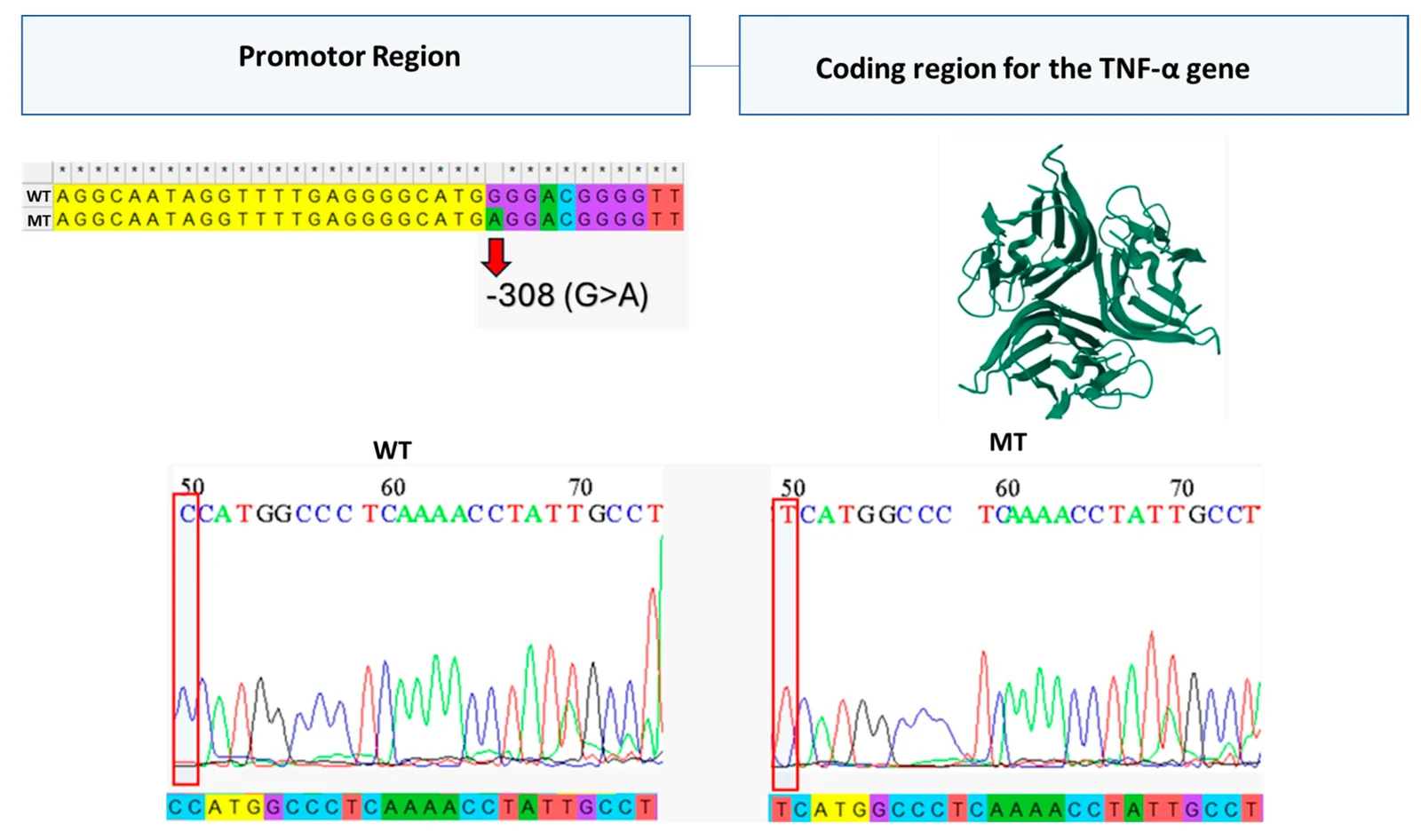
Schematic representation of the *TNF-α* -308 G>A promoter polymorphism. The figure shows the location of the -308 G>A variant relative to the forward primer sequence (FproTNF, highlighted in yellow), indicating the nucleotide substitution at position -308. A schematic of the gene region and the three-dimensional structure of *TNF-α* are also shown. At the bottom, representative chromatograms obtained from reverse primer sequencing illustrate the nucleotide position corresponding to the GG genotype and the GA genotype, highlighting the exact location of the polymorphic site. This bioinformatic workflow was used for the identification of the *TNF-α* -308 G>A polymorphism in the 92 patients analyzed.

**Figure 3:**
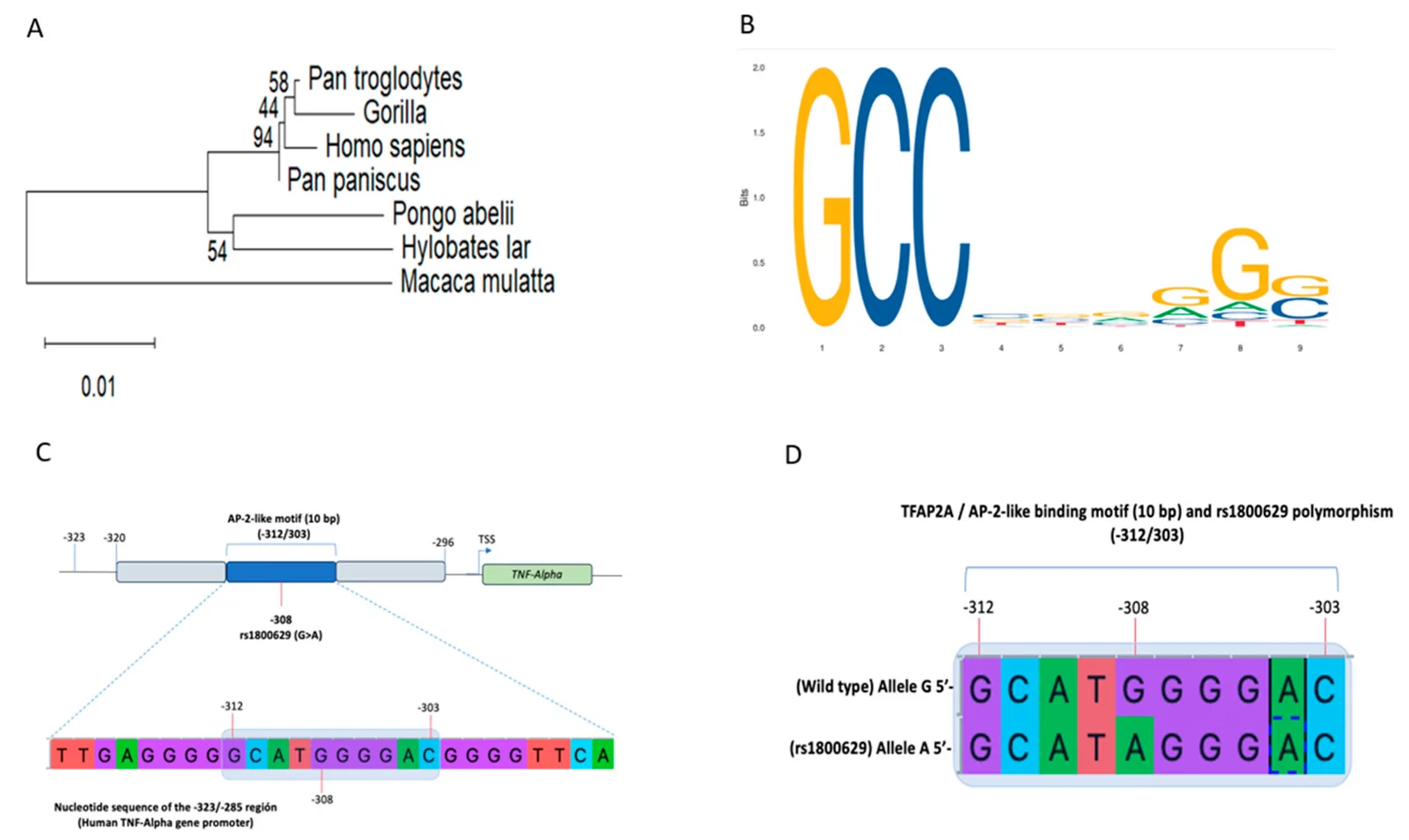
Evolutionary conservation of the *TNF-α* gene promoter and the impact of the rs1800629 polymorphism on the TFAP2A transcription factor recognition site. (**A**) of the figure shows a phylogenetic tree analyzing the evolutionary relationship among *TNF-α* gene promoter sequences across the following species: *Homo sapiens*, *Pan troglodytes*, *Gorilla*, *Pan paniscus*, *Pongo abelii*, *Hylobates lar*, and *Macaca mulatta*. (**B**) shows the AP-2 motif found in the (-312/-303) region of the gene promoter; this sequence is predicted bioinformatically as a potential binding site for the transcription factor TFAP2A. (**C**,**D**) show the location of the AP-2 motif and the position of the rs1800629 polymorphism (-308 G/A *TNF-α* gene promoter) within recognition sequence of the TFAP2A transcription factor.

A 107 base pair fragment of the *TNF-α* gene promoter was successfully sequenced in all 92 patients analyzed. The sequences obtained using forward and reverse sequencing were edited, and a consensus sequence was successfully obtained. The patient sequences were compared with the reference sequence for the *TNF-α* gene promoter, and the nucleotide at position (-308) was identified (Figure 2). The following results were obtained: among the 92 patients, 86 (93.5%) carried the GG genotype, five (5.4%) the GA genotype, and one (1.1%) the AA genotype. The frequency of the G allele was 96.2%, while the A allele frequency was 3.8%. The exact Hardy–Weinberg test showed that the genotype distribution was consistent with HWE (*p* = 0.112), indicating no significant deviation from the expected genetic equilibrium (Table 4 and Table 5). The distribution of the *TNF-α* -308 G>A variant among the patient groups was as follows: severe dengue (17 non-carriers and two A allele carriers), dengue with warning signs (41 non-carriers and two A allele carriers), and dengue without warning signs (28 non-carriers and two A allele carriers). Regarding genotype distribution, five patients presented the GA genotype (two patients with DWOS, one patient with DWWS, and two patients with SD). The homozygous AA genotype was identified in only one patient, who belonged to the DWWS group. Overall, the proportion of A allele carriers (GA/AA) was 10.5% in the SD group, 4.7% in the DWWS group, and 6.7% in the DWOS group. Pairwise Firth penalized logistic regression models, adjusted for age and primary/secondary infection status, were used to evaluate the association between *TNF-α* -308 mutant carrier status (GA/AA vs. GG) and dengue severity. Mutant allele carriers had higher odds of severe dengue compared with patients with dengue without warning signs (aOR = 1.85; 95% CI: 0.26–13.39; *p* = 0.522) and with patients with dengue with warning signs (aOR = 1.97; 95% CI: 0.24–15.07; *p* = 0.509). In the comparison between dengue with warning signs and dengue without warning signs, the adjusted OR was 0.71 (95% CI: 0.10–4.97; *p* = 0.715). None of the pairwise associations reached statistical significance, and the wide confidence intervals indicated substantial imprecision in the estimates. In the sensitivity analysis combining DWOS and DWWS as the non-severe reference group, the association remained in the same direction (aOR = 2.25; 95% CI: 0.35–11.69; *p* = 0.362), although the estimate remained imprecise and non-significant (Table 6). The *TNF-α* -308 G/A polymorphism was identified in two of 19 patients with severe dengue (10.5%) and in four of 73 patients without severe dengue (5.5%). Based on these observed frequencies, the estimated post hoc statistical power of the study was 11.3%. This value indicates a limited ability to detect statistically significant differences between groups given the sample size and the low frequency of the mutant allele. Analysis of the evolutionary conservation of the promoter for the gene encoding *TNF-α* yielded the following results: promoter sequences from *Homo sapiens*, *Pan troglodytes*, *Gorilla*, *Pan paniscus*, *Pongo abelii*, *Hylobates lar*, and *Macaca mulatta* ranged from 704 to 1183 base pairs. The human promoter sequence was 1133 base pairs long. The human promoter showed high similarity to *Pan paniscus* (0.002943 nucleotide substitutions per site) and high genetic divergence from *Macaca mulatta* (0.059075 nucleotide substitutions per site). Subsequently, a bioinformatic analysis was conducted to identify transcription factor recognition sites, revealing 132 putative sites for the factors TFAP2A, Runx1, Arnt, and AHR-ARNT heterodimer. The polymorphism was located at the AP-2-like recognition site (-312/-303) at position -308, which is predicted to be a binding site for the transcription factor TFAP2A (Figure 3).

## 4. Discussion

Dengue fever remains one of the most important vector-borne diseases and a major public health challenge in Colombia. According to the National Public Health Surveillance System (SIVIGILA), 266,090 dengue cases were reported nationwide in 2025, including 15,692 cases from the Antioquia Department. Within the Urabá subregion, the highest disease burden was concentrated in the municipalities of Apartadó, Carepa, Turbo, and Chigorodó. During 2026, up to epidemiological week 26, 132,503 dengue cases had already been reported in Colombia, demonstrating the continued endemic transmission of the virus. Although the number of reported cases in Antioquia was lower during this period, dengue remained widely distributed throughout the Urabá municipalities, confirming the persistent circulation of DENV in this region. These epidemiological data underscore the substantial public health impact of dengue in both Colombia and the Urabá subregion and highlight the need to better understand the factors associated with disease progression and severity. In this context, the present study included 92 laboratory-confirmed dengue patients from four municipalities of Urabá (Apartadó, Turbo, Carepa, and Chigorodó), providing valuable clinical and molecular information from an endemic region where dengue continues to represent a significant healthcare burden. The epidemiological information was obtained from the official database of the Colombian National Institute of Health (INS) through the SIVIGILA surveillance platform https://portalsivigila.ins.gov.co/ (accessed on 26 September 2026).

Severe dengue remains a major public health challenge in Colombia due to its capacity to cause substantial morbidity and mortality. Surveillance data from the Colombian National Institute of Health indicate the continuous occurrence of severe dengue cases in Antioquia and the Urabá subregion, highlighting the endemic nature of the disease and the need for improved strategies to identify individuals at increased risk of clinical deterioration. In addition, dengue-associated deaths continue to be reported annually, emphasizing the importance of developing reliable biomarkers for predicting disease severity and preventing fatal outcomes. In this context, our study included 19 patients with severe dengue and 43 patients with dengue with warnings signs, providing novel clinical and molecular data from an endemic region of Colombia where severe forms of the disease remain a significant healthcare concern. The data were consulted on the official website of the National Institute of Health (INS) of Colombia, through the SIVIGILA application https://portalsivigila.ins.gov.co/ (accessed on 26 September 2026).

This study focused on the molecular detection of the *TNF-α* -308 G/A promoter polymorphism and its potential relationship with the development of severe dengue in patients from the Urabá subregion of Antioquia, Colombia. Analysis of the demographic characteristics of the 92 dengue patients, including sex, place of residence, health insurance status, and educational level, revealed no statistically significant differences among the DWOS, DWWS, and SD groups (Table 1). Although these findings suggest that demographic variables were similarly distributed across the study groups, the relatively limited sample size may have reduced the ability to detect subtle associations. Nevertheless, sociodemographic factors remain important determinants of dengue transmission and disease burden. Previous studies have shown that poverty, inadequate housing conditions, limited access to safe drinking water, high population density, poor sanitation, urban marginalization, and insufficient knowledge of disease prevention contribute to increased exposure to *Aedes* mosquitoes and a higher risk of dengue infection [30,31,32]. Furthermore, several investigations have reported associations between social vulnerability indicators, including rural residence, restricted access to healthcare services, low educational attainment, poverty, and other structural inequalities, and adverse dengue outcomes, including severe clinical manifestations and mortality [33,34,35]. Although such relationships were not identified in the present study, these factors may still influence disease outcomes through delayed healthcare-seeking behavior, reduced access to timely medical intervention, and increased vulnerability to complications. Therefore, larger population-based studies are needed to further evaluate the contribution of sociodemographic determinants to dengue severity in endemic regions such as Urabá.

Regarding the clinical manifestations, dengue patients are classified into three clinical groups according to the World Health Organization (WHO) [5] and Centers for Disease Control and Prevention (CDC) [36]: dengue without (DWOS), dengue with warnings signs (DWWS), and severe dengue (DS). The main warning signs difference is the progression of the illness: the first group presents mild febrile symptoms, the second group shows signs of risk of complications, and the third group develops severe bleeding, shock, or organ failure [5,36].

Dengue without warnings signs (DWOS) is typically characterized by an acute febrile syndrome accompanied by symptoms such as headache, retro orbital pain, myalgia, arthralgia, and skin manifestations including maculopapular rash, while patients generally remain hemodynamically stable and exhibit no evidence of plasma leakage, severe bleeding, or organ dysfunction [5,36]. In the present study, 30 patients were classified as DWOS, representing 32.6% of all cases. The most frequently reported manifestations included fatigue, myalgia, dizziness, abdominal pain, arthralgia, pruritus, and exanthem. Among these, myalgia and exanthem showed statistically significant differences compared with the DWWS and SD groups, suggesting that these manifestations were more strongly associated with the mild clinical presentation of dengue. Myalgia is a hallmark symptom of uncomplicated dengue and reflects the systemic inflammatory response induced by viral replication and cytokine release, whereas exanthem is commonly associated with immune activation and cutaneous vascular involvement during the acute phase of infection. Although 10% of patients presented comorbidities, 16.7% had evidence of secondary infection, and 30% presented coinfections, none developed warnings signs or progressed to severe dengue. These findings suggest that, despite the presence of additional clinical risk factors in some individuals, the overall clinical course remained mild and self-limiting. Therefore, the DWOS group was characterized by a predominance of constitutional and musculoskeletal symptoms, preservation of clinical stability, and the absence of the vascular, hemorrhagic, or organ-related complications that define the more severe forms of dengue.

Dengue with warnings signs (DWWS) is characterized by the appearance of clinical manifestations that indicate an increased risk of progression to severe disease, including persistent abdominal pain, recurrent vomiting, fluid accumulation, mucosal bleeding, lethargy or irritability, and hepatomegaly [5,36]. Patients presenting these warnings signs require close clinical monitoring and, in most cases, hospitalization, as they may rapidly develop severe complications associated with plasma leakage, hemorrhage, or organ dysfunction [5,36]. In the present study, 43 patients were classified as DWWS, representing 46.7% of all cases. These patients presented many of the symptoms observed in the DWOS group, including retro orbital pain, fatigue, dizziness, arthralgia, and pruritus; however, additional manifestations associated with disease progression were also identified, such as fluid accumulation, ascites, irritability, hepatomegaly, splenomegaly, and hematemesis. The presence of these findings is clinically relevant because they reflect increasing vascular permeability, activation of inflammatory pathways, and early involvement of target organs, which are recognized hallmarks of disease worsening. Notably, retro orbital pain, fatigue, dizziness, arthralgia, and pruritus were among the most frequent manifestations and showed statistically significant differences when compared with the DWOS and SD groups. These symptoms may represent a stage of heightened systemic inflammation preceding the onset of severe vascular and hemorrhagic complications. Furthermore, the occurrence of warnings signs such as fluid accumulation, hepatomegaly, and hematemesis underscores the importance of early clinical recognition and intervention, as timely management during this stage may prevent progression to severe dengue. Overall, the DWWS group exhibited an intermediate clinical phenotype between uncomplicated and severe dengue, characterized by increasing inflammatory activity and the early development of pathological processes associated with severe disease.

Severe dengue (SD) is characterized by life threatening complications resulting from profound endothelial dysfunction, plasma leakage, severe hemorrhage, and multiorgan involvement, frequently requiring intensive care, intravenous fluid resuscitation, and continuous clinical monitoring [5,36]. In the present study, 19 patients were classified as SD, representing 20.7% of all cases. Although these patients shared several manifestations with the DWOS and DWWS groups, they exhibited a significantly higher frequency of symptoms associated with advanced disease progression, including abdominal pain, nausea, fluid accumulation, ascites, irritability, hepatomegaly, splenomegaly, and hematemesis. These manifestations showed statistically significant differences compared with the other patient groups and are considered key indicators of severe clinical deterioration. Fluid accumulation and ascites are direct consequences of increased vascular permeability and plasma leakage, which represent central pathogenic mechanisms of severe dengue. Similarly, hepatomegaly and splenomegaly reflect systemic inflammation and organ involvement, while hematemesis indicates significant hemostatic disturbances and an increased risk of major hemorrhagic complications. The high frequency of abdominal pain, irritability, and gastrointestinal manifestations further supports the presence of substantial inflammatory activation and disease severity. Collectively, these findings demonstrate that patients in the SD group exhibited clear clinical evidence of vascular, hematological, and organ dysfunction, consistent with the pathophysiological processes underlying severe dengue and its increased risk of morbidity and mortality.

Laboratory findings in dengue patients vary according to severity: patients without warnings signs show mild alterations, those with warnings signs present more marked hematological and biochemical changes, and severe cases show multiorgan involvement with critical values [5,36]. Laboratory findings in dengue without warnings signs (DWOS) are usually as follows: blood cell count (mild leukopenia, and moderate thrombocytopenia), hematocrit (within normal ranges or with minimal variations), transaminases (slight elevation ALT/AST), and normal coagulation tests (no evidence of plasma leakage) [5,36]. In the present investigation, the patients belonging to DWOS group showed on average: a slight increase in respiratory rate (RR), a slight decrease in hemoglobin (Hb), a slight increase in basophils, an increase in aspartate aminotransferase (AST), and a slight decrease in creatinine. Clinically, these findings are consistent with the mild systemic inflammatory response that characterizes dengue without warnings signs. The slight increase in respiratory rate may reflect the febrile state and increased metabolic demand associated with acute viral infection rather than respiratory compromise. The mild reduction in hemoglobin values could be related to individual baseline variability or hemodilution, since patients in this group did not exhibit evidence of plasma leakage or hemoconcentration. Elevated AST levels are frequently observed during uncomplicated dengue and suggest a transient involvement of hepatic tissue or muscle cells caused by viral replication and the host inflammatory response. Likewise, the slight increase in basophil counts may reflect activation of immune and inflammatory pathways during the acute phase of infection. The lower creatinine levels observed in this group are unlikely to be clinically relevant and indicate preserved renal function, contrasting with the renal impairment commonly reported in severe dengue. Overall, the laboratory profile of DWOS patients supports the presence of a self-limited infection with mild hematological and biochemical alterations, without evidence of significant vascular leakage, coagulation abnormalities, or organ dysfunction.

Laboratory findings in dengue with warnings signs (DWWS) are usually the following: blood cell count (marked thrombocytopenia < 100,000/µL), hematocrit (rapid increase, reflecting hemoconcentration due to plasma leakage), transaminases (significant elevation AST/ALT > 3 times the normal value), hypoalbuminemia, electrolyte disturbances, evidence of fluid accumulation (ascites, and pleural effusion) [5,36]. In our study, the patients belonging to DWWS group showed on average: a slight increase in respiratory rate (RR), a decrease in platelets, an increase in aspartate aminotransferase (AST), a slight decrease in creatinine, and a slight increase in activated partial thromboplastin time (aPTT). Clinically, these findings are consistent with the intermediate stage of dengue severity, in which systemic inflammation, endothelial activation, and early plasma leakage begin to develop. The slight increase in respiratory rate may reflect the combined effects of fever, increased metabolic demand, and the initial consequences of plasma leakage, even in the absence of overt respiratory compromise. Thrombocytopenia was one of the most relevant findings in this group and is considered a hallmark of disease progression, resulting from bone marrow suppression, immune-mediated platelet destruction, and increased peripheral consumption. The elevated AST levels suggest hepatic involvement, a common feature of dengue infection that may arise from direct viral effects on hepatocytes, immune-mediated tissue injury, or transient hypoperfusion. The mild prolongation of aPTT indicates the early development of coagulation abnormalities and consumption of clotting factors, which have been associated with increased vascular permeability and a higher risk of hemorrhagic manifestations. In contrast, creatinine concentrations remained slightly decreased, indicating preserved renal function and the absence of significant kidney injury at this stage of the disease. Overall, the laboratory profile observed in the DWWS group reflects a transition between uncomplicated dengue and severe disease, characterized by progressive hematological, hepatic, and hemostatic alterations that may precede the development of plasma leakage, hemorrhage, and organ dysfunction.

Laboratory findings in severe dengue (SD) are usually the following: blood cell count (severe thrombocytopenia < 50,000/µL), hematocrit (very high, associated with hypovolemic shock), transaminases (critical values > 1000 IU/L, indicating severe liver damage), coagulation (prolongation of prothrombin time (PT) and activated partial thromboplastin time (aPTT), consumption of coagulation factors), metabolic acidosis, elevated creatinine and urea (renal failure), hypocalcemia, and elevated lactate [5,36]. In the present investigation, patients in the SD group exhibited: a slight increase in pulse pressure, a slight increase in heart rate (HR), a slight increase in respiratory rate (RR), a decrease in eosinophils (Eos), an increase in aspartate aminotransferase (AST), an increase in prothrombin time (PT), and an increase in activated partial thromboplastin time (aPTT). Clinically, these findings are consistent with the profound systemic involvement that characterizes severe dengue. The slight increases in heart rate and respiratory rate may represent compensatory physiological responses to intravascular volume depletion, systemic inflammation, and increased metabolic demand associated with severe infection. Although pulse pressure did not show marked abnormalities, hemodynamic alterations in severe dengue are frequently related to endothelial dysfunction and plasma leakage, which may precede the development of shock. The decrease in eosinophil counts is compatible with the intense inflammatory response and stress associated leukocyte redistribution observed during severe viral infections. Elevated AST levels indicate hepatic involvement, one of the most common manifestations of severe dengue, resulting from a combination of direct viral effects, immune-mediated injury, and tissue hypoperfusion secondary to vascular leakage. In addition, the prolongation of both PT and aPTT reflects activation and dysregulation of the coagulation system, likely associated with consumption of clotting factors, liver dysfunction, thrombocytopenia, and endothelial damage. These abnormalities contribute to the increased risk of hemorrhagic events and are consistent with the higher frequency of hematemesis observed in this patient group. Overall, the laboratory profile of the SD patients supports the presence of advanced pathological processes involving inflammatory activation, hepatic injury, hemostatic imbalance, and circulatory dysfunction, which are recognized hallmarks of severe dengue and major determinants of morbidity and mortality.

When comparing the patient groups, statistically significant differences were observed in several laboratory parameters associated with disease severity. Patients with severe dengue (SD) exhibited significantly lower platelet counts, eosinophil percentages, and basophil percentages compared to the DWWS and DWOS groups. In contrast, hematocrit, white blood cell counts, creatinine, blood urea nitrogen (BUN), and activated partial thromboplastin time (aPTT) were significantly higher in SD patients. These findings are clinically relevant because they reflect key pathophysiological processes associated with severe dengue, including plasma leakage, hemoconcentration, coagulopathy, systemic inflammation, and organ dysfunction. The markedly reduced platelet counts and prolonged aPTT support the presence of hemostatic impairment and an increased risk of bleeding, while elevated hematocrit values are indicative of vascular permeability and intravascular volume loss. Similarly, increased creatinine and BUN levels suggest renal involvement, whereas leukocyte elevation may reflect a more intense inflammatory response. Patients in the DWWS group generally displayed intermediate laboratory values between those observed in the DWOS and SD groups, suggesting a progressive deterioration of hematological, coagulation, and biochemical parameters as disease severity increases. Collectively, these results support the existence of a continuum of pathological changes during dengue progression, ranging from mild systemic inflammation in uncomplicated cases to substantial vascular, hemostatic, and multiorgan involvement in severe disease.

The *TNF-α* -308 A allele was infrequent in the study population, and mutant allele carriers were proportionally more frequent among patients with severe dengue. However, after adjustment for age and primary/secondary infection status using Firth penalized logistic regression, the associations between mutant carrier status and dengue severity remained non-significant and highly imprecise, as reflected by the wide confidence intervals. The sensitivity analysis comparing severe with non-severe dengue showed a similar direction of association but with substantial uncertainty. Therefore, these findings should be interpreted as exploratory and do not provide conclusive evidence that the *TNF-α* -308 G/A polymorphism is associated with increased dengue severity. The genotype distribution did not show a statistically significant deviation from Hardy–Weinberg equilibrium (*p* = 0.112); however, this finding should not be interpreted as evidence of absence of population stratification or other potential sources of bias.

The genetic analysis of the relationship between polymorphisms and the presence of infection remains inconclusive in the literature [37,38]. Some previous reviews reported no significant association between -308 *TNF-α* G>A and -238 *TNF-α* G>A with the risk of dengue hemorrhagic fever (DHF) [37]. However, the findings suggest limited and inconsistent evidence regarding the association of -308 *TNF-α*/-238 *TNF-α* G>A polymorphisms with the risk of developing severe dengue infection [38]. Previous literature indicates variable effects of the G/G and G/A genotypes of the -308 *TNF-α* SNP on susceptibility to dengue across Asian and Brazilian populations [22,39,40]. Some studies from Latin America have suggested that the -308 SNP G/G genotype is associated with an increased risk of hemorrhagic manifestations in patients with dengue. In this regard, Fernández-Mestre and collaborators [41] indicated that the significant increase of the -308 *TNF-α* G/A allele among patients with dengue fever (DF) with hemorrhagic manifestations compared to patients with DF only indicates that the first patient group is genetically predisposed to express higher levels of *TNF-α*.

Studies from Mexico and a systematic review from Latin America have reported heterogeneous associations between the *TNF-α* -308 G/A polymorphism and dengue-related clinical phenotypes [42,43]. Some studies have suggested a possible association between the A allele and more severe manifestations; however, the available evidence remains inconsistent across populations. In the present study, mutant allele carriers were proportionally more frequent among patients with severe dengue, and the adjusted point estimates for severe dengue compared with DWOS and DWWS were greater than 1. Nevertheless, these associations were not statistically significant and were characterized by wide confidence intervals, indicating substantial uncertainty in the magnitude and direction of the effect. Therefore, our findings should be considered exploratory and cannot confirm an association between the *TNF-α* -308 G/A polymorphism and dengue severity. Larger, adequately powered studies in genetically diverse populations are needed to clarify this relationship. The potential biological relevance of this polymorphism remains plausible because *TNF-α* has been implicated in endothelial dysfunction, increased vascular permeability, and inflammatory responses associated with severe dengue. However, *TNF-α* expression or circulating cytokine levels were not measured in the present study; therefore, a functional mechanism cannot be inferred from these data.

The transition from a systemic inflammatory response to dengue shock syndrome remains poorly understood, although epidemiological evidence underscores the substantial role of genetic factors in disease susceptibility. Specifically, several human leukocyte antigen (HLA) class I and II alleles have been associated with the onset of dengue hemorrhagic fever (DHF). Additionally, variations in genes such as tumor necrosis factor alpha (*TNF-α*), Fcγ receptor, vitamin D receptor, CTLA-4, and transforming growth factor beta (TGF-β) have been implicated in developing severe dengue manifestations [23].

The biological roles of certain cytokines and soluble factors in the development of hemorrhage are crucial for understanding dengue disease progression. It has been previously demonstrated that cytokines such as *TNF-α*, IFN-γ, IL-6, and IL-10 are elevated in severe dengue cases, with *TNF-α* notably associated with increased endothelial permeability and exacerbation of symptoms [21,44,45,46,47]. A significant correlation between soluble *TNF-α* levels and thrombocytopenia has also been reported [48]. Specifically, the -308 *TNF-α* G/A allele, linked to heightened cytokine production, appears more frequently in patients with dengue hemorrhagic fever (DHF). The capacity of *TNF-α* to enhance endothelial permeability indicates its potential role in the pathogenesis of DHF, as evidenced by a mouse model where elevated *TNF-α* levels were correlated with endothelial cell (EC) apoptosis and hemorrhage [42,45,49]. Additionally, *TNF-α* is implicated in mediating pathology through the lysis of infected cells and facilitating CD8+ T-cell cytotoxicity [50].

In this study, the *TNF-α* -308 G>A polymorphism was identified in the promoter region, consistent with previous literature linking this variant to altered transcriptional activity of *TNF-α* gene [41,46]. Various studies have presented differing associations of this allele with specific dengue phenotypes ranging from being protective against DHF/DSS in Asia and Latin America, showing no association in Mexico, providing marginal protection against DF in Brazil under a dominant model, to being identified as a risk factor for DHF in Venezuela, Cuba, and other parts of Latin America [39,40,41,42,44]. The global frequency of the TNF rs1800629 (-308G>A) A allele varies among population databases. In the 1000 Genomes Project (https://www.internationalgenome.org/), the A allele has a global frequency of approximately 9.0%, whereas higher frequencies have been reported in gnomAD (approximately 13.8%) (https://gnomad.broadinstitute.org/) and TOPMed (13.5%) (https://topmed.nhlbi.nih.gov/). These differences may reflect variation in sample size, population composition, sequencing datasets, and ancestry representation across databases. The TNF rs1800629 (-308G>A) polymorphism is located within the -323/-285 promoter region, which contains a 10 base pairs sequence homologous to the consensus binding motif of activator protein-2 (AP-2). This region has been shown to influence transcriptional activity and to interact with nuclear protein complexes [51]. Furthermore, the -308G>A substitution has been reported to modify allele-specific transcription factor binding and to produce differences in TNF promoter activity between the two alleles [52,53]. In silico analyses have additionally predicted that rs1800629 may alter transcription factor recognition sites, including motifs associated with SP1, USF, ATF3, and CCNT2 [54]. However, these predictions should be interpreted as potential regulatory effects rather than direct evidence of transcription factor binding. Importantly, although the -323/-285 region contains an AP-2-like sequence, subsequent experimental analysis demonstrated that AP-2, MZF-1, and SP1 were not components of the specific DNA–protein complex showing preferential binding to the -308A allele [53]. Therefore, TFAP2A may be considered a candidate transcription factor for the regulation of *TNF-α* transcription at this promoter region; however, direct allele-specific binding of TFAP2A to rs1800629 has not been established. Experimental validation using approaches such as allele-specific EMSA and ChIP-qPCR would be necessary to determine whether TFAP2A binds to TNF promoter and whether the -308G>A substitution modifies this interaction. Complementary promoter reporter assays comparing the −-08G and −308A alleles would further determine whether differences in transcription factor binding translate into functional changes in promoter activity [51,52,53,54].

The observations from some studies highlight a relationship between disease severity and secondary infections, through the analysis of single nucleotide polymorphisms (SNPs) in various genes, including *TNF-α*, IL-6, IFN-γ, IL-10, and TGFβ1 [21,46]. In our study, assessing this relationship proved challenging due to the limited variability, given that nearly 90% of patients experienced a primary infection. Despite this limitation, it remains reasonable to hypothesize about the interplay between inflammatory responses induced by secondary infections and cytokine level alterations, potentially indicative of genetic variations.

The immune response plays a critical role in combatting DENV infection, with both innate and adaptive immunity significantly influencing disease outcomes [21,46]. Within the context of the innate immune response, *TNF-α* is notably pivotal, as its elevated levels have been correlated with increased severity of dengue and thrombocytopenia [22,50]. Prior studies have highlighted the significance of high viral loads, macrophage infiltration, and *TNF-α* production in local tissues as key factors leading to hemorrhage in dengue patients [43,50]. Moreover, models have demonstrated that the intradermal inoculation of high DENV titers predisposes endothelial cells to *TNF-α*-induced apoptosis, facilitating endothelial damage and the onset of hemorrhage. This illustrates the integral role of the innate immune response in the pathogenesis of dengue hemorrhage, a relationship mirrored in our study’s observations of severe dengue signs such as fluid accumulation, hematemesis, hepatomegaly, and splenomegaly.

Several limitations should be considered when interpreting these findings. First, the relatively small sample size and the low frequency of the *TNF-α* -308 mutant allele resulted in sparse data and limited statistical power (11.3%), leading to wide confidence intervals and imprecise effect estimates. Although Firth penalized logistic regression was used to reduce small sample bias, the limited number of mutant allele carriers restricted the number of covariates that could be reliably included in the adjusted models. After adjustment for age and primary/secondary infection status, the point estimates for severe dengue remained greater than 1 in the pairwise comparisons; however, none of the associations reached statistical significance, and the confidence intervals were wide. Similarly, the sensitivity analysis comparing severe dengue with the combined non-severe group showed an effect estimate in the same direction but remained imprecise and non-significant. Therefore, these findings should be considered exploratory rather than confirmatory.

Second, residual confounding cannot be excluded. DENV serotype could not be incorporated into the adjusted models because this information was unavailable for a substantial proportion of participants, and other potentially relevant factors, such as delay in healthcare seeking, were not systematically available. In addition, the geographical distribution of the sample limits its representativeness: 65.2% of participants were recruited from Apartadó, and patients were included from only four of the eleven municipalities of the Urabá subregion. Consequently, the sample may not fully capture the demographic and genetic diversity of the entire Urabá population, and the findings should not be generalized beyond the study population. Future multicenter studies with larger samples and broader geographical representation are warranted. Finally, because all participants had laboratory-confirmed dengue, this study addresses the association of the *TNF-α* -308 polymorphism with disease severity rather than susceptibility to DENV infection. Studies including an appropriately selected non-dengue comparison group would be needed to investigate genetic susceptibility to infection.

Third, the present study focused on a single *TNF-α* promoter variant and did not evaluate additional cytokine or chemokine pathways. Future studies should incorporate variants and/or expression markers from complementary inflammatory pathways, including CCL2/MCP-1 and CXCL8/IL-8, to better characterize the host inflammatory genetic architecture associated with dengue severity.

## 5. Conclusions

This study provides a molecular characterization of the *TNF-α* -308 G/A promoter polymorphism in laboratory-confirmed dengue patients recruited from four municipalities of the Urabá subregion of Antioquia, Colombia. The clinical and laboratory findings demonstrated a progressive increase in disease severity from dengue without warnings signs to severe dengue, characterized by the development of vascular, hematological, coagulation, and organ-related abnormalities. Molecular analysis identified the *TNF-α* -308 G/A polymorphism in a small proportion of patients, with a higher frequency observed among severe dengue cases. Although no statistically significant association was detected between the polymorphism and dengue severity, severe dengue patients exhibited higher odds of carrying the mutant allele compared with the other clinical groups. These findings support the hypothesis that host genetic factors, particularly variants involved in the regulation of the inflammatory response, may contribute to the clinical outcome of dengue infection. Furthermore, the results reinforce the biological relevance of *TNF-α* in dengue immunopathogenesis. Future studies involving larger sample sizes, healthy control populations, multicenter recruitment, and additional immune response genes are required to validate these observations and improve our understanding of the genetic determinants associated with severe dengue in endemic regions of Latin America. Additionally, it is necessary to evaluate complementary inflammatory pathways, including CCL2/MCP-1 and CXCL8/IL-8, to clarify the contribution of host genetic variation to dengue severity.

## Figures and Tables

**Table 1:** Demographic characteristics of the dengue patients. The table shows the demographic information, place of residence, type of health insurance and educational level of the 92 patients included in the research.

Characteristics	DWOS (n = 30)	DWWS (n = 43)	SD (n = 19)	*p* Value ^a^
	n (%)	n (%)	n (%)	
Demographic				
Age (Mean/s)	13.9/15.4	11.1/8.5	16.2/15.8	
Age (Median/IR)	10.0/11.0	10.0/7.0	8.0/21.0	0.921 ^d^
Sex				
Women	13 (43.3)	19 (44.2)	9 (47.4)	0.960
Men	17 (56.7)	24 (55.8)	10 (52.6)	
Place of residence				
Urban	16 (53.3)	25 (58.1)	16 (84.2)	0.074
Rural	14 (46.7)	18 (41.9)	3 (15.8)	
Health Insurance type				
Contributory	17 (56.7)	24 (55.8)	15 (78.9)	0.262
Subsidized	12 (40.0)	19 (44.2)	4 (21.1)	
Uninsured	1 (3.3)	-	-	
Education level *				
Daycare	3 (10.0)	4 (9.3)	2 (40.0)	0.354 ^b^
Elementary school	13 (43.3)	24 (55.8)	1 (20.0)	
High school	11 (36.7)	11 (25.6)	1 (20.0)	
No formal education	3 (10.0)	3 (7.0)	1 (20.0)	
University	-	1 (2.3)	-	

Notes: * Missing: education level 14. IR: interquartile range. ^a^: chi-square test of association. ^b^: Fisher test. ^d^: Kruskal test. SD: Severe dengue. DWWS: dengue with warnings signs. DWOS: dengue without warnings signs.

**Table 2:** Clinical characteristics of the dengue patients. The table shows the comorbidity, infection, coinfection, and symptoms and signs of the 92 patients included in the research.

Characteristics	DWOS (n = 30)	DWWS (n = 43)	SD (n = 19)	*p* Value ^a^
	n (%)	n (%)	n (%)	
Comorbidity *				
Yes	3 (10.0)	6 (14.3)	5 (26.3)	0.312 ^b^
No	27 (90.0)	36 (85.7)	14 (73.7)	
Infection *				
Primary	25 (83.3)	38 (95.0)	14 (73.7)	0.052 ^b^
Secondary	5 (16.7)	2 (5.0)	5 (26.3)	
Coinfection *				
Yes	9 (30.0)	8 (19.5)	5 (26.3)	0.584
No	21 (70.0)	33 (80.5)	14 (73.7)	
Symptoms and signs				
Retro orbital pain	9 (30.0)	25 (58.1)	3 (15.8)	**0.003 ^b^**
Abdominal pain	18 (60.0)	36 (83.7)	17 (89.5)	**0.021**
Nausea	7 (23.3)	37 (86.0)	17 (89.5)	**0.000**
Fatigue	24 (80.0)	40 (93.0)	11 (57.9)	**0.004**
Dizziness	9 (30.0)	20 (46.5)	2 (10.5)	**0.019**
Myalgia *	27 (90.0)	37 (88.1)	8 (42.1)	**0.000**
Arthralgia *	24 (80.0)	36 (85.7)	9 (47.4)	**0.004**
Exanthem *	16 (53.3)	22 (52.4)	3 (15.8)	**0.016**
Pruritus *	14 (46.7)	23 (54.8)	2 (10.5)	**0.005**
Edema of extremities *	1 (3.3)	5 (11.9)	2 (21.1)	0.137 ^b^
Fluid accumulation *	0.0 (0.0)	6 (14.3)	11 (57.9)	**0.000 ^b^**
Ascites *	0.0 (0.0)	5 (11.9)	7 (36.8)	**0.001 ^b^**
Irritability *	0.0 (0.0)	1 (2.4)	5 (26.3)	**0.001 ^b^**
Hepatomegaly *	0.0 (0.0)	10 (23.8)	12 (66.7)	**0.000 ^b^**
Splenomegaly *	0.0 (0.0)	2 (4.8)	5 (26.3)	**0.002 ^b^**
Hematemesis *	0.0 (0.0)	1 (2.4)	4 (21.1)	**0.009 ^b^**

Notes: * Missing: Comorbidity 1, Infection 3, Coinfection 2, Myalgia 1, Arthralgia 1, Exanthem 1, Pruritus 1, Edema of extremities 1, Fluid accumulation 1, Ascites 1, Irritability 1, Hepatomegaly 2, Splenomegaly 1, Hematemesis 1. ^a^: Chi-square test of association. ^b^: Fisher test. DWWS: dengue with warnings signs. DWOS: dengue without warnings signs. Statistically significant values are highlighted in bold.

**Table 3:** Laboratory characteristics of the dengue patients. The table shows the vital, hematological, biochemical and coagulation parameters of the 92 patients included in the study.

Characteristic	DWOS (n = 30)	DWWS (n = 43)	SD (n = 19)	*p*-Value ^d^
	Median (IR)	Median (IR)	Median (IR)	
Systolic Blood Pressure (SBP)	100.0 (5.0) N	100.0 (11.3) N	102.0 (17.0) N	0.875
Diastolic Blood Pressure (DBP)	60.0 (5.0) N	60.0 (10.0) N	62.0 (8.0) N	0.898
Mean Arterial Pressure (MAP)	73.3 (8.3) N	73.3 (11.6) N	76.7 (8.8) N	0.769
Pulse Pressure	40.0 (5.0) N	35.0 (10.0) N	41.0 (15.0) ↑	0.260
Heart Rate (HR)	97.0 (22.0) N	101.5 (25.3) N	112.0 (19.0) ↑	**0.037 ^e^**
Respiratory Rate (RR)	22.0 (5.0) ↑	22.0 (4.0) ↑	20.0 (4.5) ↑	0.565
Oxygen Saturation (SatO2) *	98.0 (2.0) N	98.0 (1.0) N	98.0 (0.8) N	0.063
Hemoglobin *	11.7 (2.6) ↓	12.6 (2.3) N	13.8 (3.0) N	**0.036**
Hematocrit *	36.9 (5.9) N	39.0 (5.4) N	40.5 (9.5) N	**0.046**
Platelet Count *	200,500.0 (146,250.0) N	103,000.0 (87,000.0) ↓	64,000.0 (45,000.0) ↓	**<0.001**
White Blood Cell Count (WBC) *	6345.0 (4267.5) N	4705.0 (1985.0) N	8190.0 (4530.0) N	**0.004**
Neutrophils *	42.1 (42.6) N	49.9 (34.5) N	51.8 (22.3) N	0.092 **^e^**
Lymphocytes *	41.8 (29.3) N	36.4 (23.9) N	34.2 (18.0) N	0.279 **^e^**
Eosinophils *	4.9 (4.6) N	1.4 (2.7) N	0.9 (3.7) ↓	**0.001**
Basophils *	1.2 (1.1) ↑	0.8 (0.6) N	0.8 (0.6) N	**0.034**
Aspartate Aminotransferase (AST) *	61.5 (78.1) ↑	87.1 (120.0) ↑	105.7 (69.8)	0.672
Alanine Aminotransferase (ALT) *	24.0 (72.3) N	41.3 (84.3) N	44.1 (59.4) N	0.901
C Reactive Protein (CRP) *	1.0 (1.0) N	0.5 (2.1) N	3.3 (11.0) N	0.206
Creatinine *	0.7 (0.3) ↓	0.5 (0.1) ↓	0.8 (0.5) N	**0.001**
Blood Urea Nitrogen (BUN) *	6.8 (2.1) N	9.0 (5.5) N	14.0 (14.3) N	**0.011**
Prothrombin Time (PT) *	10.0 (2.7) N	11.4 (2.2) N	14.3 (7.6) ↑	**0.099**
Activated Partial Thromboplastin Time (aPTT) *	33.6 (3.6) N	32.0 (10.8) ↑	47.2 (22.6) ↑	**0.025 ^e^**

Notes: * Number of missing data: Oxygen Saturation (1), Hb (1), Hct (1), Plt (1), WBC (1), Neut (1), Lymph (2), Eos (3), Baso (5), AST (3), ALT (3), CRP (48), Cr (4), BUN (7), PT (8), aPTT (8). IR: Interquartile Range. ^d^: Kruskal test. ^e^: ANOVA test. SD: Severe dengue. DWWS: Dengue with warnings signs. DWOS: Dengue without warnings signs. Normal values (N). Increased values (↑). Decreased values (↓). Statistically significant values are highlighted in bold.

**Table 4:** Sequencing and genotype assignment results for the *TNF-α* -308 G>A promoter polymorphism in the 92 patients analyzed.

Patient	Nucleotide (-308 Position)	Genotype	Patient	Nucleotide	Genotype
P1	G/G	Wild-type	P47	G/G	Wild-type
P2	G/G	Wild-type	P48	G/G	Wild-type
P3	G/G	Wild-type	P49	G/G	Wild-type
P4	G/G	Wild-type	P50	G/G	Wild-type
P5	G/A	Heterozygous	P51	G/G	Wild-type
P6	G/G	Wild-type	P52	G/G	Wild-type
P7	G/G	Wild-type	P53	G/G	Wild-type
P8	G/G	Wild-type	P54	G/G	Wild-type
P9	G/G	Wild-type	P55	G/A	Heterozygous
P10	G/G	Wild-type	P56	G/G	Wild-type
P11	G/G	Wild-type	P57	G/G	Wild-type
P12	G/G	Wild-type	P58	G/G	Wild-type
P13	G/G	Wild-type	P59	G/G	Wild-type
P14	G/A	Heterozygous	P60	G/G	Wild-type
P15	G/G	Wild-type	P61	G/G	Wild-type
P16	G/G	Wild-type	P62	G/A	Heterozygous
P17	G/G	Wild-type	P63	G/G	Wild-type
P18	G/G	Wild-type	P64	G/G	Wild-type
P19	G/G	Wild-type	P65	G/G	Wild-type
P20	G/G	Wild-type	P66	G/G	Wild-type
P21	G/A	Heterozygous	P67	G/G	Wild-type
P22	A/A	Homozygous	P68	G/G	Wild-type
P23	G/G	Wild-type	P69	G/G	Wild-type
P24	G/G	Wild-type	P70	G/G	Wild-type
P25	G/G	Wild-type	P71	G/G	Wild-type
P26	G/G	Wild-type	P72	G/G	Wild-type
P27	G/G	Wild-type	P73	G/G	Wild-type
P28	G/G	Wild-type	P74	G/G	Wild-type
P29	G/G	Wild-type	P75	G/G	Wild-type
P30	G/G	Wild-type	P76	G/G	Wild-type
P31	G/G	Wild-type	P77	G/G	Wild-type
P32	G/G	Wild-type	P78	G/G	Wild-type
P33	G/G	Wild-type	P79	G/G	Wild-type
P34	G/G	Wild-type	P80	G/G	Wild-type
P35	G/G	Wild-type	P81	G/G	Wild-type
P36	G/G	Wild-type	P82	G/G	Wild-type
P37	G/G	Wild-type	P83	G/G	Wild-type
P38	G/G	Wild-type	P84	G/G	Wild-type
P39	G/G	Wild-type	P85	G/G	Wild-type
P40	G/G	Wild-type	P86	G/G	Wild-type
P41	G/G	Wild-type	P87	G/G	Wild-type
P42	G/G	Wild-type	P88	G/G	Wild-type
P43	G/G	Wild-type	P89	G/G	Wild-type
P44	G/G	Wild-type	P90	G/G	Wild-type
P45	G/G	Wild-type	P91	G/G	Wild-type
P46	G/G	Wild-type	P92	G/G	Wild-type

**Table 5:** Allele frequencies and Hardy–Weinberg equilibrium analysis of the *TNF-α* -308 G/A polymorphism in the study population (n = 92).

Parameter	Value
Total individuals (N)	92
GG genotype, n (%)	86 (93.5%)
GA genotype, n (%)	5 (5.4%)
AA genotype, n (%)	1 (1.1%)
G allele frequency (*p*)	0.962 (96.2%)
A allele frequency (q)	0.038 (3.8%)
Expected GG (*p*^2^ × N)	85.13
Expected GA (2*pq* × N)	6.73
Expected AA (*q*^2^ × N)	0.13
Exact Hardy–Weinberg test (*p*-value)	0.112
Hardy–Weinberg equilibrium	Yes

Notes: Allele frequencies were estimated by direct counting. Hardy–Weinberg equilibrium (HWE) was assessed using the exact Hardy–Weinberg test. A *p*-value > 0.05 indicates no significant deviation from equilibrium. The observed genotype distribution was consistent with HWE (*p* = 0.112).

**Table 6:** Distribution of the *TNF-α* −308 G/A polymorphism and adjusted associations with dengue severity.

	DWOS (n = 30)	DWWS (n = 43)	SD (n = 19)
Panel A. Genotype and mutant allele carrier distribution, n (%)			
Carrier status			
Non-carrier (GG)	28 (93.3)	41 (95.3)	17 (89.5)
Carrier (GA/AA)	2 (6.7)	2 (4.7)	2 (10.5)
Genotype			
GG	28 (93.3)	41 (95.3)	17 (89.5)
GA	2 (6.7)	1 (2.3)	2 (10.5)
AA	0 (0.0)	1 (2.3)	0 (0.0)
Panel B. Pairwise association between mutant carrier status and dengue severity			
	aOR	95% CI	*p*-value
SD vs. DWOS	1.85	0.26–13.39	0.522
SD vs. DWWS	1.97	0.24–15.07	0.509
DWWS vs. DWOS	0.71	0.10–4.97	0.715
Sensitivity analysis			
SD vs. non-severe dengue (DWOS + DWWS)	2.25	0.35–11.69	0.362

Notes: SD: severe dengue. DWWS: dengue with warnings signs. DWOS: dengue without warnings signs. aOR: adjusted odds ratio. CI: confidence interval. Mutant carrier status was defined as GA/AA versus GG. Adjusted estimates were obtained using Firth penalized logistic regression controlling for age and primary/secondary infection status. In the sensitivity analysis, DWOS and DWWS were combined into a single non-severe dengue reference group.

## Data Availability

The *TNF-α* gene promoter sequence in the 92 patients was deposited in the NCBI GeneBank database under the following accession numbers: PZ897907, PZ897908, PZ897909, PZ897910, PZ897911, PZ897912, PZ897913, PZ897914, PZ897915, PZ897916, PZ897917, PZ897918, PZ897919, PZ897920, PZ897921, PZ897922, PZ897923, PZ897924, PZ897925, PZ897926, PZ897927, PZ897928, PZ897929, PZ897930, PZ897931, PZ897932, PZ897933, PZ897934, PZ897935, PZ897936, PZ897937, PZ897938, PZ897939, PZ897940, PZ897941, PZ897942, PZ897943, PZ897944, PZ897945, PZ897946, PZ897947, PZ897948, PZ897949, PZ897950, PZ897951, PZ897952, PZ897953, PZ897954, PZ897955, PZ897956, PZ897957, PZ897958, PZ897959, PZ897960, PZ897961, PZ897962, PZ897963, PZ897964, PZ897965, PZ897966, PZ897967, PZ897968, PZ897969, PZ897970, PZ897971, PZ897972, PZ897973, PZ897974, PZ897975, PZ897976, PZ897977, PZ897978, PZ897979, PZ897980, PZ897981, PZ897982, PZ897983, PZ897984, PZ897985, PZ897986, PZ897987, PZ897988, PZ897989, PZ897990, PZ897991, PZ897992, PZ897993, PZ897994, PZ897995, PZ897996, PZ897997, PZ897998.

## Supplementary Materials

The following supporting information can be downloaded at https://www.mdpi.com/article/10.3390/microorganisms14102354/s1, Table S1: Database of patients with dengue and polymorphism in TNF alpha; Figure S1: *TNF-α* Gene Promoter Amplification and Visualization.
