# Supplementary material for: Molecular Detection of the -308 G/A Polymorphism in the Tumor Necrosis Factor Alpha (TNFα) Gene Promoter and Its Relationship with the Development of Severe Dengue in Patients from Urabá Subregion, Antioquia-Colombia
Source: Microorganisms. 2026 Oct 14;14(10):2354. doi: 10.3390/microorganisms14102354 (PMC13707562; doi:10.3390/microorganisms14102354)
Supplement: Supplementary file 1 [file microorganisms-14-02354-s001.zip › Supplementary Figure S1.pdf]

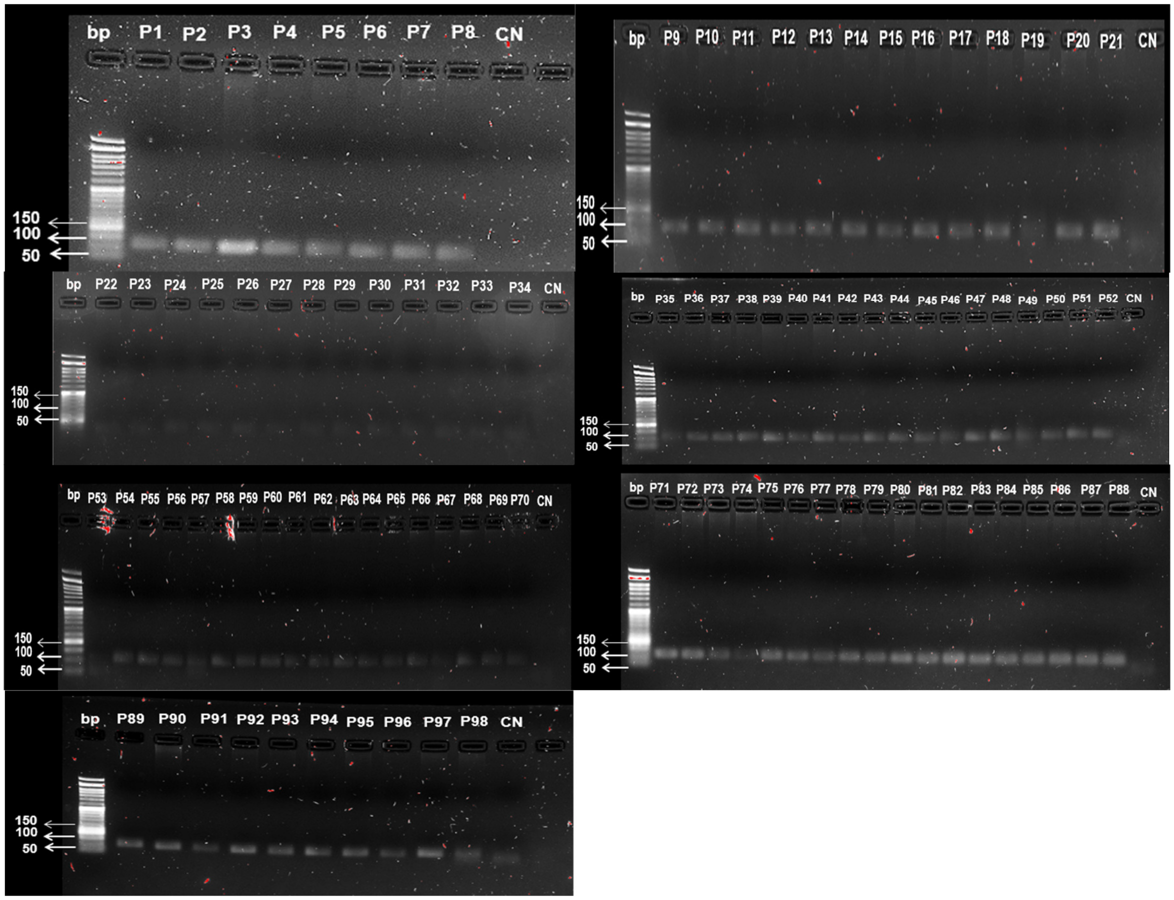

Supplementary Figure S1. *TNF-α* Gene Promoter Amplification and Visualization. The agarose gel shows the amplification of a 107 base pair fragment in the promoter region of the *TNF-α* gene. A molecular weight marker (50 base pair scale) was used to determine the size of the amplification products. Numbers (P1–P92) are the amplification products of the promoter region from the 92 patients included in the study. Samples (P93–P98) are dengue negative samples used as positive amplification controls. Negative controls from the PCR reactions were included in each gel to rule out possible contamination.
